# Gestational diabetes mellitus is associated with increased pro-migratory activation of vascular endothelial growth factor receptor 2 and reduced expression of vascular endothelial growth factor receptor 1

**DOI:** 10.1371/journal.pone.0182509

**Published:** 2017-08-17

**Authors:** Felipe Troncoso, Jesenia Acurio, Kurt Herlitz, Claudio Aguayo, Patricio Bertoglia, Enrique Guzman-Gutierrez, Marco Loyola, Marcelo Gonzalez, Meriem Rezgaoui, Gernot Desoye, Carlos Escudero

**Affiliations:** 1 Vascular Physiology Laboratory, Group of Investigation in Tumor Angiogenesis (GIANT), Chillán, Chile; 2 Department of Basic Sciences, Universidad del Bío-Bío, Chillán, Chile; 3 Department of Clinical Biochemistry and Immunology, Faculty of Pharmacy, University of Concepción, Concepción, Chile; 4 Group of Research and Innovation in Vascular Health (GRIVAS Health), Chillan, Chile; 5 Obstetric and Gynecology Department, Hospital Clinico Herminda Martin, Chillán, Chile; 6 Universidad Católica de la Santísima Concepción, Concepción, Chile; 7 Facultad de Ciencias de la Salud, Universidad San Sebastián, Concepción, Chile; 8 Vascular Physiology Laboratory, Department of Physiology, Faculty of Biological Sciences, Universidad de Concepción, Concepción, Chile; 9 Department of Obstetrics and Gynaecology, Medical University of Graz, Graz, Austria; Centro Cardiologico Monzino, ITALY

## Abstract

Placentas from gestational diabetes mellitus (GDM) are often hypervascularized; however, participation of vascular endothelial growth factor (VEGF) and its receptors in this placental adaptation is unclear. We aimed to test whether changes in phosphorylation of tyrosine 951 or tyrosine 1175 (pY951 or pY1175) of the vascular endothelial growth factor receptor 2 (KDR) are associated with the proangiogenic state observed in placentas from GDM. We obtained placental samples from women with normal pregnancies (n = 24) or GDM (n = 18). We measured the relative expression of markers for endothelial cell number (CD31, CD34), VEGF, vascular endothelial growth factor receptor 1 (Flt-1), KDR, pY951 and pY1175 of KDR in placental homogenate. Immunohistochemistry of placental blood vessels were performed using CD34. Proliferation and migration of human umbilical vein endothelial cells (HUVEC) obtained from normal pregnancy and GDM were determined in absence or presence of conditioned medium (CM) harvested from GDM or normoglycemic HUVEC cultures. GDM was associated with more CD31 and CD34 protein compared to normal pregnancy. High number, but reduced area of placental blood vessels was found in GDM. Reduced Flt-1 levels (mRNA and protein) are associated with reduced KDR mRNA, but higher KDR protein levels in placentas from GDM. No significant changes in Y951-or Y1175-phosphorylation of KDR in placentas from GDM were found. GDM did not alter proliferation of HUVECs, but enhanced migration. Conditioned medium harvested from GDM HUVEC cultures enhanced KDR protein amount, tube formation capacity and cell migration in HUVEC isolated from normoglycemic pregnancies. The data indicate that GDM is associated with reduced expression of Flt-1 but high pro-migratory activation of KDR reflecting a proangiogenic state in GDM.

## Introduction

Gestational diabetes mellitus (GDM) affects at least 1 in 10 pregnant woman worldwide [1, 2] and accounts for a range of adverse perinatal outcomes including, among others, excessive fetal fat accretion (‘macrosomia’), fetal hypoglycemia, requirement of neonatal intensive care and neonatal mortality [2–5]. In addition, GDM causes not only short-term complications in the newborn, but is also associated with an elevated risk for chronic conditions such as cardiovascular disease, obesity and diabetes [6, 7].

The placenta is an essential organ for fetal growth and development. It mounts adaptive responses to changes in the intrauterine environment, which occur during normal and also pathological pregnancies [8]. Indeed, compared to normal pregnancies, placentas from GDM exhibit enhanced vascularization [9, 10]. This may appear counterintuitive in a situation of maternal nutritional oversupply, but reflects the increased oxygen demand of the fetus, because of the insulin-stimulated enhanced fetal aerobic metabolism [10]. The mechanisms underlying enhanced angiogenesis have not been fully elucidated.

Vascular endothelial growth factor (VEGF) is one of the main regulators of angiogenesis [11, 12]. It activates the three tyrosine kinase receptors VEGF receptor 1 (VEGFR1 or Fms-like tyrosine kinase 1, Flt-1), VEGFR2 (or kinase insert domain receptor, KDR) and VEGFR3 [13, 14]. Of these VEGFR-1/Flt-1 and VEGFR-2/KDR are expressed most prominently in vascular endothelial cells, where Flt-1, different from KDR, has a poor kinase activity, but an elevated capacity to bind VEGF. Thus Flt-1 has been considered as a negative regulator of KDR activation during angiogenesis [11]. On the other hand, KDR has potent tyrosine kinase activity and is, therefore, regarded as main transducer of VEGF effects on angiogenesis including proliferation, migration and tube formation of endothelial cells [11, 12]. At least five major phosphorylation sites on KDR have been described, which can activate different intracellular signaling pathways [11]. Among these autophosphorylation sites, phosphorylation on tyrosine 951 (pY951) has been related to cell migration [15], while pY1175 has been mainly related to cell proliferation [11]. Therefore, identification of the sites of KDR phosphorylation may help suggest, which particular processes involved in angiogenesis are enhanced during VEGF activation.

Several studies have reported placental levels of VEGF and its receptors in GDM, but with differing results likely due to selection of patients, differences in diagnostic criteria of GDM and methodological differences [6, 16–19]. However, there are no reports regarding functional aspects of KDR in GDM, which may help to dissect the mechanisms underlying placental hypervascularization in GDM. Therefore, we hypothesize that GDM is associated with elevated placental proangiogengenic markers and changes in tyrosine phosphorylation of KDR

## Patients and methods

### Patients

Pregnant women, who were attended by members of the Obstetric and Gynecology Department of the Herminda Martin Clinical Hospital, Chillán, Chile, for their delivery, were included in this study. Informed consent was obtained from each participant. Placentas were collected after delivery from 24 full-term normal or 18 full-term gestational diabetic pregnancies. Women were classified as having (1) normal pregnancy (maternal blood pressure <140/90 mm Hg, basal glycaemia <90mg/dL during entire pregnancy and no medical complications), (2) Criteria for gestational diabetes mellitus (GDM) diagnosis, which was defined as glycaemia >90 mg/dL (i.e., overnight starvation) and >140 mg/dL at 2 hours after an oral glucose load (75 g) at 22–24 weeks of gestation [20]. Investigation was performed according to the principles expressed in the Declaration of Helsinki. The Ethical Committee of the Universidad del Bío-Bío approved this cohort study.

### Sampling of placentas

Within 20 minutes after delivery, placentas were weighed and examined macroscopically to exclude clots, fibrosis, or areas of infarction. Four random full-depth samples of the placentas were then taken from each tissue as previously reported [21]. Samples were immersed in liquid nitrogen and maintained at -80°C until used for mRNA and protein extraction. The four samples were pooled for analysis.

### Human umbilical vein endothelial cell (HUVEC) culture

In a subset of samples (14 each group), endothelial cells were isolated from the human umbilical vein (HUVEC) by digestion with collagenase (0.25 mg/mL) and then cultured (37°C, 5% CO_2_) in medium 199 (M199) as previously described [22]. All experiments were performed in duplicate, after overnight serum deprivation. Cells were used in passage 2. In culture endothelial cells develop the characteristic cobblestone phenotype [23].

In some experiments HUVEC isolated from either normal or GDM pregnancies were cultured in M199 without serum and conditioned medium (CM) was harvested after 48 hours. After collection, CM was centrifuged at 10,000 *x g* at 4°C for 10 minutes, sterilized by filtration through a 0.22 μm pore-size filter and maintained at -80°C until use within two weeks.

### Microscopic analysis and immunocytochemistry

KDR protein was identified by immunocytochemistry in HUVEC isolated from normal pregnancies and HUVEC exposed to conditioned medium (CM) from GDM HUVEC (see above) by using a commercial kit (Vector Laboratories, MI, USA) following the manufacturer’s protocol. Briefly, after treatment HUVECs were fixed in 4% paraformaldehyde prepared in phosphate buffer (PBS, (mM): NaCl 13.7, KCl 2.7, Na_2_HPO_4_ 0.9, KH_2_PO_4_ 1.8, pH 7.4, 4°C) for 20 minutes. After blocking unspecific binding using 5% bovine serum albumin (BSA) in blocking buffer solution (PBS/Tween 1%), the cells were incubated overnight with primary KDR antibody (Cell Signaling Technology, Boston, USA) (Clone 55B11, dilution 1:200 v/v). Antigen-antibody reaction was further revealed by HRP-conjugated secondary antibody (Vector Laboratories, MI, USA).

Images were taken by a camera and the intensity of the immunocytochemical staining was semi-quantified using ImageJ software (National Institutes of Health, Bethesda, MD) [24]. Values are expressed as the ratio between the area of brown stain and the total area of the reference field. All samples for digital and microscopic analyses were blinded to the operator.

### Western blot

Placental homogenates were prepared in an Ultra-Turrax homogenizer (Daihan Scientific, Seoul, South Korea). HUVEC mRNA and protein were extracted from primary cultures of normal and GDM pregnancies. Both extractions were prepared in lysis buffer (Tris HCL, pH 8, 20 mM; NaCl 137 mM; EDTA 2 mM; glycerol 10%, Nonidet P-40 1%) and protease inhibitor cocktail (Thermo Scientific, CA, USA). Samples were centrifuged at 14,000 *x g* at 4°C for 10 minutes. Proteins (70 μg) from supernatant were separated by SDS-PAGE (10%), transferred to nitrocellulose membranes, and probed with primary antibodies for CD31 (Abcam, Cambridge, UK; ab54211, dilution 1:1000 v/v), CD34 (Abcam ab8158, dilution 1:2500 v/v), VEGF (Abcam ab1316, 1:2000 v/v); Flt-1 (Santa Cruz Biotechnology, TX, USA; sc-316, dilution 1:1000 v/v); KDR (Cell Signaling Technology, MA, USA; #2479, dilution 1:1000 v/v), phospho-Y951-KDR (Cell Signaling Technology; #4991, dilution 1:1000 v/v) and β-actin (Sigma-Aldrich, MO, USA; clone AC-74, dilution 1:15000 v/v). A horseradish peroxidase-conjugated secondary antibody was used for visualization (rabbit, Thermo Scientific, CA, USA; mouse, Sigma-Aldrich, MO, USA). Bands on gels were scanned and images quantified by ImageJ 1.48 software (National Institute of Health, USA) as described previously [22].

### Microscopic analysis and immunohistochemistry

Microscopic analysis and immunohistochemistry analysis was performed as previously describe for our group with minor modifications [21]. Briefly, paraffin-embedded tissue sections were cut into 5-mm slices to use for immunodetection of CD34 (Abcam ab8536, dilution 1:250 v/v) using a commercial kit (Vector Laboratories, Burlingame, CA). Antigen-antibody reaction was visualized by diaminobenzidine reaction, and densitometry was performed using Image-Pro Plus software (Media Cybernetics, Rockville, MD) in two random pictures from each preparation. This approach, allowed us to count the number of blood vessels and chorionic villi. Also, we measure the area of every vessel and its corresponding villi. Values are expressed as number of vessels per villi and average area of those vessels per villi. Two different blinded observers analyzed those images.

### ELISA for VEGF and KDR tyrosine phosphorylation

The PathScan Phospho-VEGFR-2 (Tyr-1175) sandwich ELISA kit (Cell signaling Technology) was used to measure tyrosine-1175 phosphorylation of KDR (pKDR) in total placental homogenate (300 μg). VEGF concentrations in conditioned culture medium (200 μg of total protein) were quantified by ELISA (R&D Systems, # DVE00). Both ELISA were performed following the manufacturer’s instructions.

### Semi quantitative PCR

RNA was isolated using TRIzol® Reagent (Life Technology, CA, USA) according to the manufactures' instructions. RNA concentration was measured in MaestroNano (Maestrogen, NV, USA). An absorption ratio of 260/280nm ~2.0 was considered as high quality RNA preparation. The RNA concentration was used to standardize the amount of RNA of each sample for cDNA synthesis. Reverse transcription were performed as previously described [25], using the SV Total RNA Isolation System (Promega, Madison, WI) according to the manufacturer’s instructions.

mRNA levels for Flt-1, KDR, VEGF, and 18S were assessed by semi-quantitative PCR in the linear phase of amplifaction (Multigene Gradient, Labnet, NJ, USA). Reactions in 20 μL were carried out, using PCR Master Mix with 1-μM primers, according to the manufacturer´s instructions. The primers and cycles used are described in Table 1. Products of the expected size were separated by electrophoresis on 1.5% agarose gels and visualized with ethidium bromide under UV light.

**Table 1:** Primers used for RT-PCR.

Protein	Sequence	Tm (melting point)	Product size	Annealing	Cycles
VEGF	F: 5- GGGCAGAATCATCACGAAGTG -3	56.1	65	56	30
	R:5- ATTGGATGGCAGTAGCTGCG -3	58.1			
Flt-1	F: 5- TCCCTTATGATGCCAGCAAGT-3	56.5	79	50	32
	R:5- CCAAAAGCCCCTCTTCCAA-3	55.3			
KDR	F: 5- CTTCGAAGCATCAGCATAAGAAACT-3	55.5	156	50	35
	R:5- TGGTCATCAGCCCACTGGAT-3	56.4			
18S	F: 5- TCAAGAACGAAAGCTGGAGG-3	54.9	500	50	25
	R:5- GGACATCTAAGGGCATCACA-3	54.3			

### Cell proliferation and migration

Cell proliferation was analyzed in absence or presence (24 h) of conditioned medium as described above by MTS assay (Roche Diagnostics, IN, USA) following the manufacturer’s instructions.

Cell migration was analyzed also in absence or presence (8 or 24 h) of conditioned medium using wound healing assay [22]. Briefly, cells were allowed to reach confluence in growth medium, and then switched to serum-free medium prior to addition of either normal or GDM-derived conditioned medium. The monolayer was wounded with a single sterile cell scraper of constant diameter. Cells were observed at 40X magnification in a phase contrast inverted microscope (Olympus, Tokyo, Japan), and six random images were taken using digital camera (MShot MD90, Guangzhou Micro-shot Technology Co., Ltd, China) immediately after wound generation and 24 hours after treatment. Cell migration was analyzed using both cell counting and the area measurement plugin from ImageJ software. Migratory area is expressed as percentage of migration in respect of denudated area.

$$M.area=(A_{0}-\frac{A_{nh}}{A_{0}})*100$$

Where *M*. *area* denotes the migratory area. *A*_*0*_ is the area at time 0, or denudated area, *A*_*nh*_ represents the area, which remained denudated after 8 or 24 hours accordingly with experimentation.

### Angiogenesis assay

HUVECs (4 x10^4^) from normal pregnancies were cultured in a 96-well plate coated with 40 μL Matrigel Basement Membrane Matrix (Corning Labware, MA, USA). Assays were performed in absence or presence (8 hours) of conditioned medium derived from GDM. Tube length was quantified using the “Angiogenesis analyzer” plugin from ImageJ software.

### Statistical analysis

Groups were compared by non-parametric analysis (Mann-Whitney test). Values are presented as median and interquartile range, or media ± S.E.M. as appropriate. *P*<0.05 is considered statistically significant. GraphPad Prism 5.00 (GraphPad Software, La Jolla, CA) was used for data and statistical analysis.

## Results

### Clinical characteristics

Forty-two pregnant women were included in the study, 24 with a normal pregnancy and 18 women with GDM (Table 2). The GDM women had higher weight, BMI in the first trimester (9 ± 1 weeks), and weight gain than non-GDM women. In addition, the number of prenatal care visit for clinical evaluation was higher (p<0.05) in women with GDM compared to controls. There were no significant differences in other clinical parameters including gestational age at delivery, systolic and diastolic blood pressure, and rate of cesarean section or newborn’s anthropometry. Placentas from GDM were heavier (P = 0.01) than those from normal pregnancy by about 100 grams. Ratio of newborn weight to placental weight was reduced (P = 0.007) in GDM compared to controls.

**Table 2:** Characteristics of women included in the study.

	Normal pregnancy (n = 24)	GDM (n = 18)
**Maternal**		
Weight at first trimester (Kg)	61.9 ± 2.3	68.8 ± 3.0*
Weight at delivery (Kg)	75.2 ± 2.2	76.6 ± 1.9
Height (cm)	156 ± 1.3	158 ± 1.9
Weight gain (Kg)	13.3 ± 4.5	7.8 ± 4.9*
Body mass index first trimester (Kg/cm2)	25.3 ± 0.9	27.9 ±1.2*
Body mass index at delivery (Kg/cm2)	30.8 ± 0.8	31.1 ± 0.9
Parity (number of gestations)	1.3 ± 0.3	1.8 ± 0.3
Prenatal care visits (#)	10.6 ± 0.6	14.9 ± 1.9*
Gestational age at delivery (wk)	39.1 ± 0.2	39.5 ± 0.2
Systolic blood pressure (mmHg)	115.3 ± 3.0	119.6 ± 1.5
Diastolic blood pressure (mmHg)	73.3 ± 2.7	70.7 ± 2.7
Cesarean section (% of women)	50	43
**Newborn**		
Sex (male/female)	14/10	8/10
Weight (g)	3607 ± 105	3655 ± 112
Height (cm)	49.6 ± 0.4	49.3 ± 0.6
Cephalic perimeter (cm)	35.1 ± 0.2	35.2 ± 0.3
**Placenta**		
Weight (g)	558 ± 26.2	658 ± 35.6**
New born weight/Placental weight	6.9 ± 0.2	5.9 ± 0.4*

Gestational diabetes mellitus (GDM).

*P<0.05, and

**P = 0.01 versus normal pregnancy

### Protein expression of endothelial markers and VEGF

**Fig 1:**
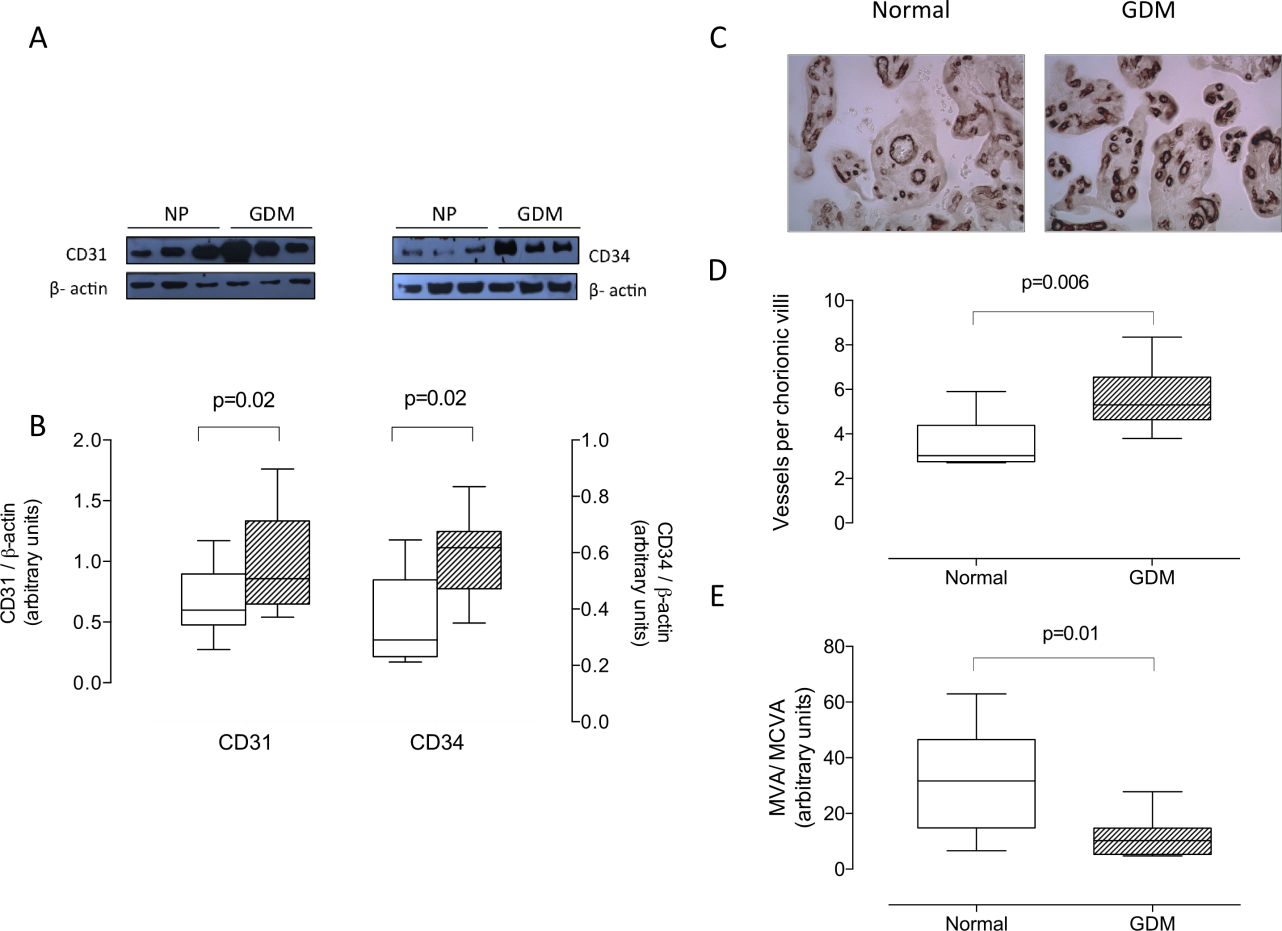
Placental CD31 and CD34 protein. Protein levels of CD31 and CD34 in total placental homogenates of normal pregnancy (NP, white bar) and gestational diabetes mellitus (GDM, hatched bar) were determined by western blot. **A)** Representative western blot for CD31 (55 kDa), CD34 (80 kDa) and β-actin (43 kDa). **B)** Densitometric analysis of CD31/ β-actin ratio (left Y-axis) and CD34/ β-actin ratio (right Y-axis). **C)** Representative images of CD34 staining in placentas from Normal and GDM. **D)** Number of vessels per villi. **E)** Mean blood vessel area (MVA)/mean chorionic villi area (MCVA) ratio. In B, n = 6–12 in each group. In D and E, n = 4–5 in each group. Magnification 40X. P-value is indicated in each graph.

Protein levels of CD31 and CD34 (Fig 1A and 1B) in placentas from GDM were higher than those in normal pregnancy. Also, number of chorionic blood vessels (CD34 positive staining) per villi was greater in GDM than normal pregnancy (5.6 ± 0.5 vs 3.5 ± 0.3 vessels per villi, respectively, P = 0.006) (Fig 1C and 1D). However, average area of those vessels was lower in GDM than normal pregnancy (-63%, P = 0.01) (Fig 1E).

**Fig 2:**
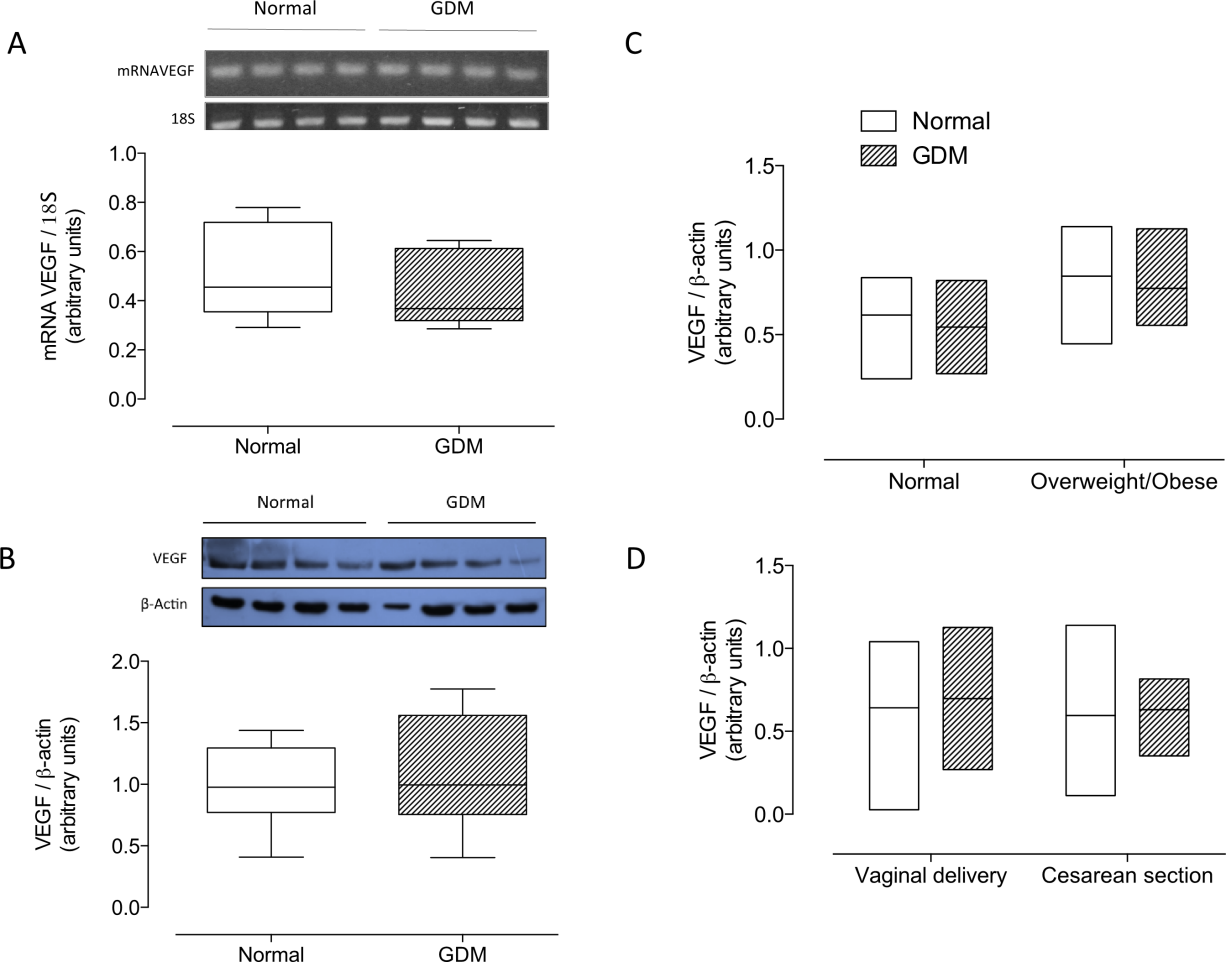
Placental VEGF. **A)** mRNA levels for VEGF in total placental homogenate from normal pregnancies (Normal, white bar) or gestational diabetes mellitus (GDM, hatched bar). Values are expressed as ratio mRNAVEGF/18S obtained by densitometric analysis. **B)** Western blot of VEGF (55 kDa) and β-actin (43 kDa). The bottom panel shows the results of densitometric scanning expressed as VEGF/β-actin ratio. **C)** Post-hoc analysis of VEGF protein levels as presented in B considering maternal body mass index (BMI) in the first trimester **D)** or delivery mode. In A, n = 8 in each group. B, n = 14–15 in each group.

Placental VEGF mRNA (Fig 2A) and protein levels (Fig 2B) did not differ between GDM and controls. Furthermore, no significant changes were observed in VEGF protein levels when data was analyzed grouping normal first trimester body mass index or considering delivery mode (Fig 2C and 2D). The protein levels of CD31, CD34 or VEGF were not significantly correlated with newborn anthropometry or placental weight (data not shown).

### Vascular endothelial growth factor receptor 1 and 2

**Fig 3:**
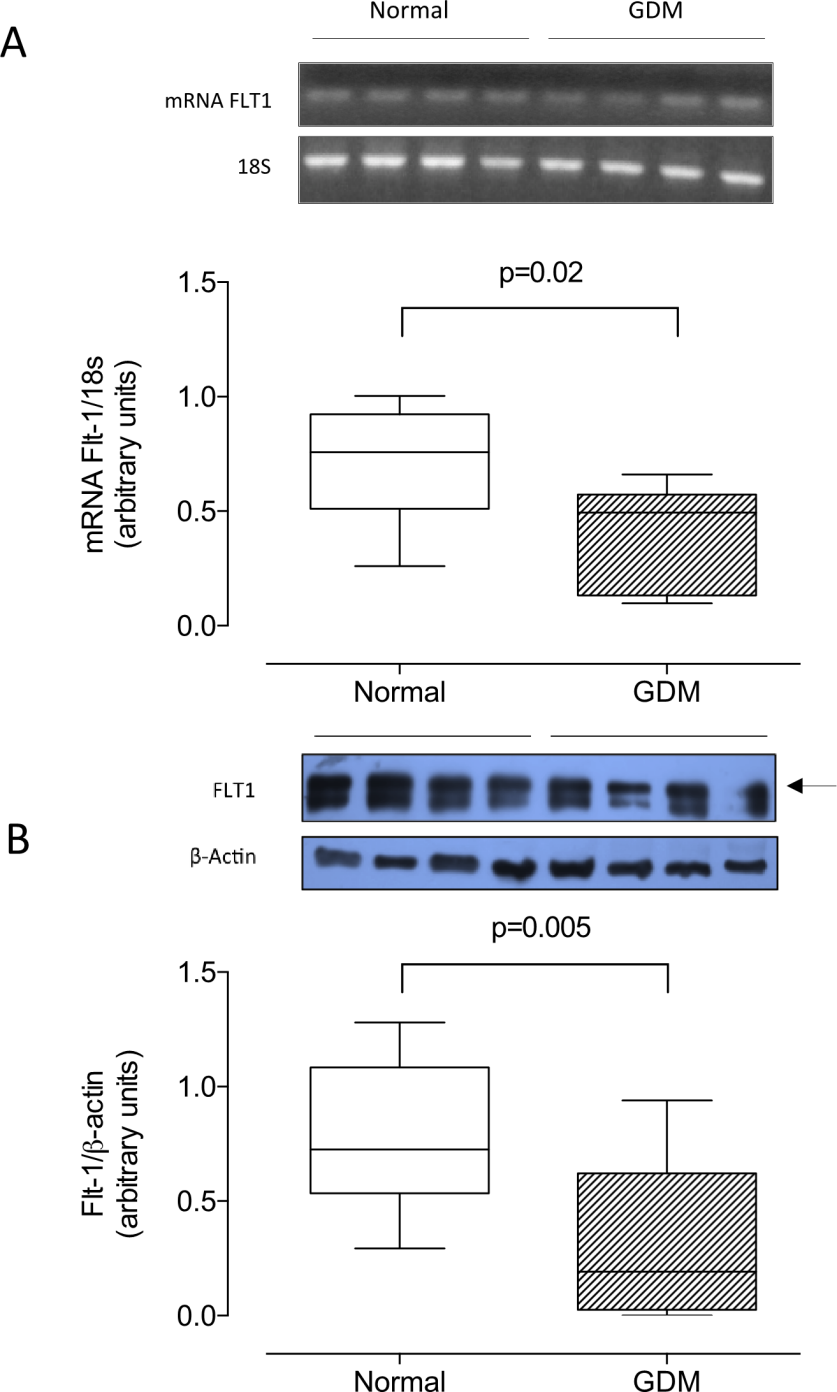
Placental Flt-1. **A)** mRNA levels for Flt-1 in total placental homogenate (n = 8 in each group) from normal pregnancies (Normal, white bar) or gestational diabetes mellitus (GDM, hatched bar). Values are expressed as ratio mRNA Flt-1/18S obtained by densitometric analysis. **B)** Western blot of Flt-1 (210 kDa) and β-actin (43 kDa). The bottom panel shows the results of densitometric scanning expressed as Flt-1/β-actin ratio; n = 15 in each group. P-value is indicated in each graph.

Compared to normal pregnancy, placentas from GDM exhibited significantly lower mRNA (-42%, Fig 3A) and protein (-59%, Fig 3B) levels of Flt-1. Differences remain significant in vaginally delivery pregnancies, but was attenuated in those delivered by cesarean section (see S1A Fig).

**Fig 4:**
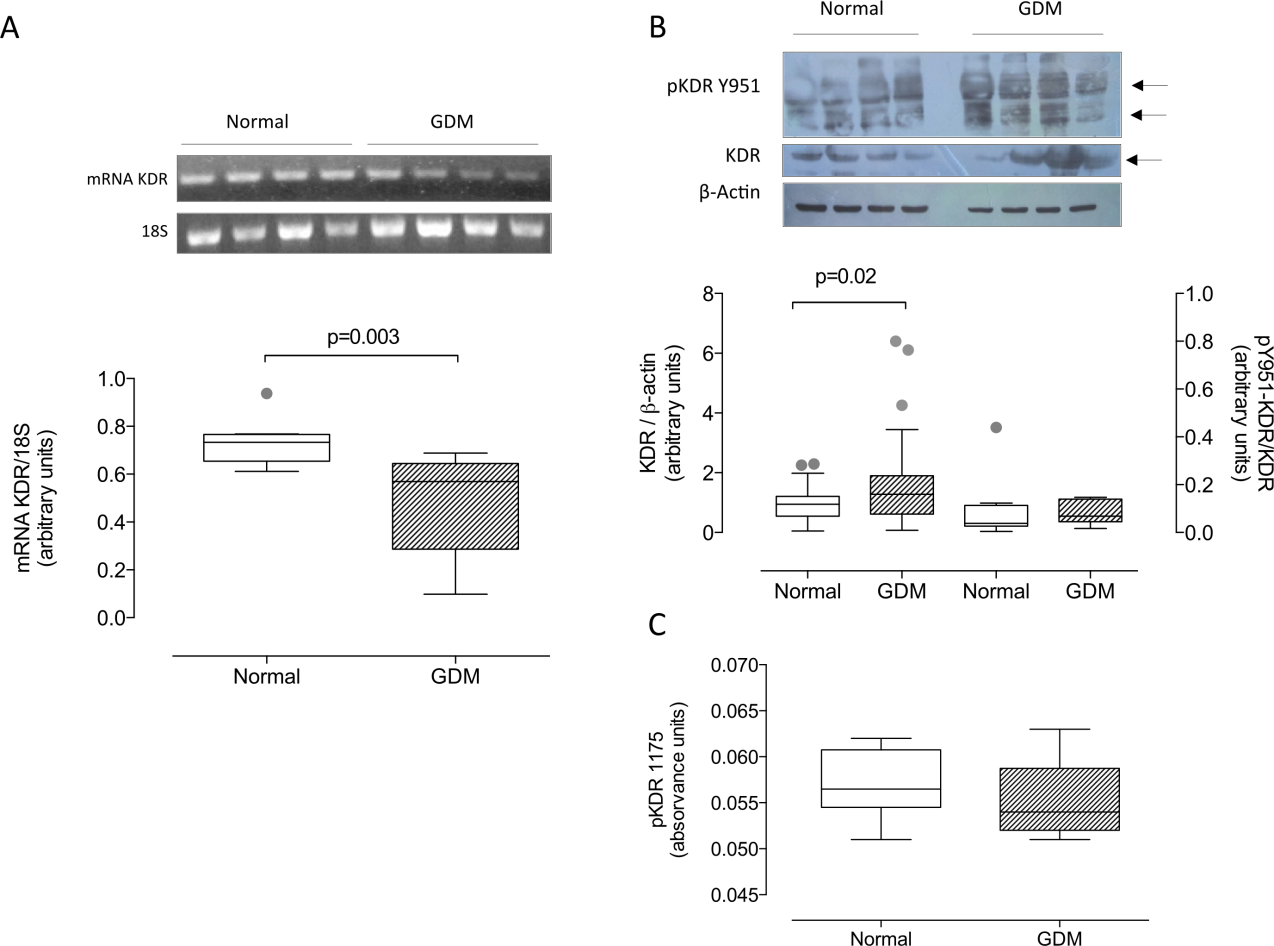
Total KDR and tyrosine 951-and tyrosine 1175 phosphorylation of KDR. **A)** Representative image of PCR for KDR and 18S in placental homogenates from normal (Normal, white bar) and gestational diabetic pregnancies (GDM, hatched bar). In the bottom panel is presented densitometic analysis of mRNA KDR/18S. **B)** Representative images of total KDR (170kDa) and tyrosine 951 phosphorylated (pY951-KDR) (70 kDa) and β-actin (43 kDa) in placenta homogenates. Chart represents densitometry of KDR/β-actin ratio and pY951-KDR/KDR. **C)** Optical units at 450 nm were used for estimating the relative amount of KDR phosphorylated in the Y1175 residue. Significant differences between groups are indicated with respective P value. In A and C, n = 8 in each group. In B, n = 18 in GDM, n = 20, for total KDR; while 8 per group for Y951. P-value is indicated in each graph. Outliners are indicated in gray.

Also KDR mRNA was lower (-34%, P = 0.003) (Fig 4A), while KDR protein levels were higher (Fig 4B) (1.7 fold, P = 0.02) in GDM placentas than in those from normal pregnancy, and were unaffected by mode of delivery (see S1B Fig).

## Differential tyrosine phosphorylation of KDR in GDM

The ratio of Y951-phosphorylated KDR (pY951-KDR) to total KDR estimated by western blot was similar in normal and GDM placentas (Fig 4B). Similar results were obtained for Y1175-phosphorylated KDR as determined by ELISA (Fig 4C).

### Cell proliferation and migration of HUVEC isolated from GDM

**Fig 5:**
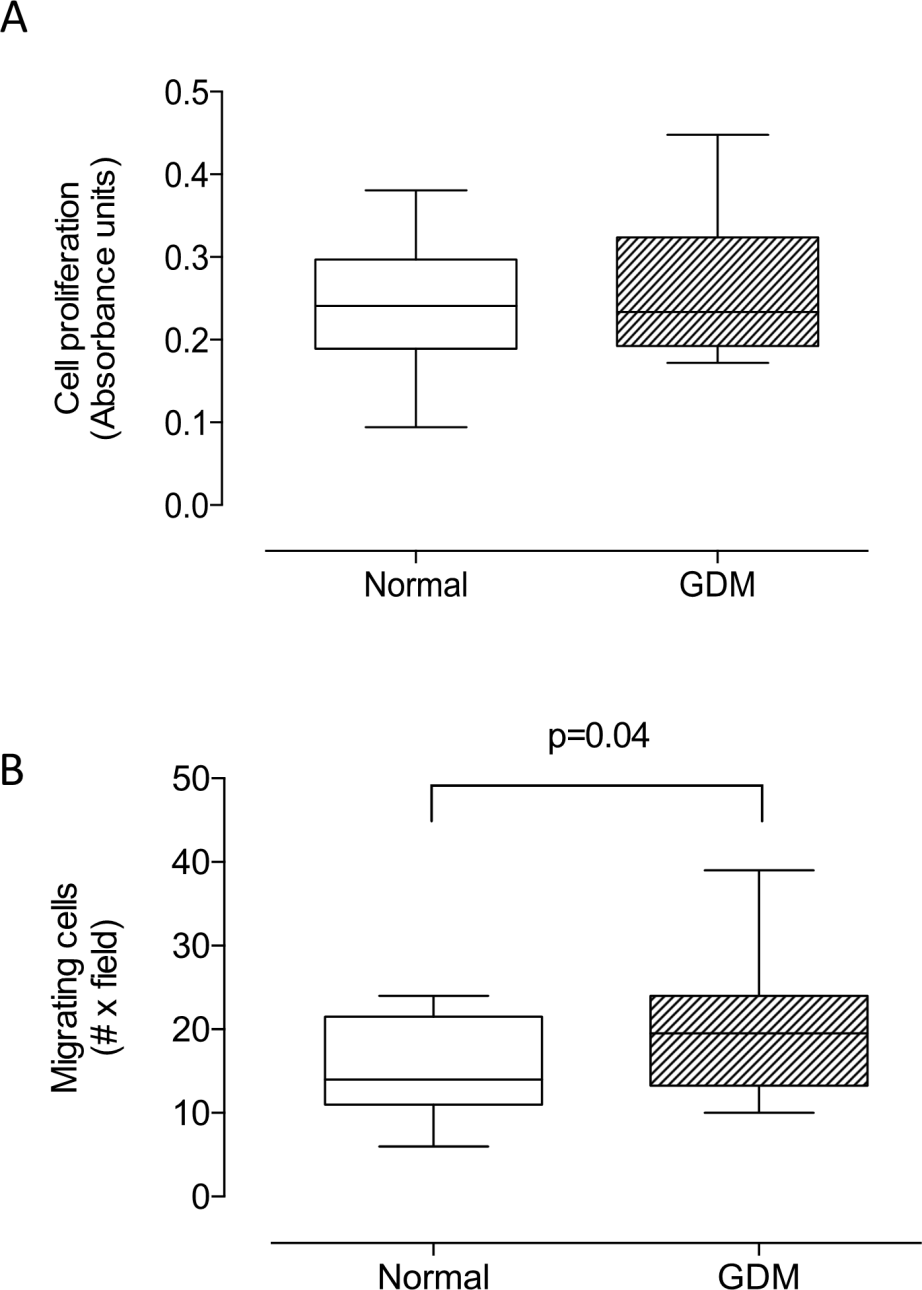
Cell proliferation and migration in HUVEC from gestational diabetes. **A)** Cell proliferation measured by MTS assay in HUVEC from normal (Normal, white bar) and gestational diabetes mellitus (GDM, hatched bar) pregnancies. **B)** Cell migration as in A. In A, n = 8–11; in B, n = 13 in each group.

Proliferation (24 h, culture) did not differ between HUVEC isolated from GDM compared to those isolated from normal pregnancy (Fig 5A).

Nevertheless, significantly more migrating cells (i.e. cells crossing the gap after 24 hours) were observed in HUVEC isolated from GDM compared to normal pregnancy (Fig 5B, 20 ± 1.1 vs 15 ± 1.1 cells per field, respectively, P = 0.04).

### Conditioned medium from cell culture of GDM promote angiogenesis in vitro

**Fig 6:**
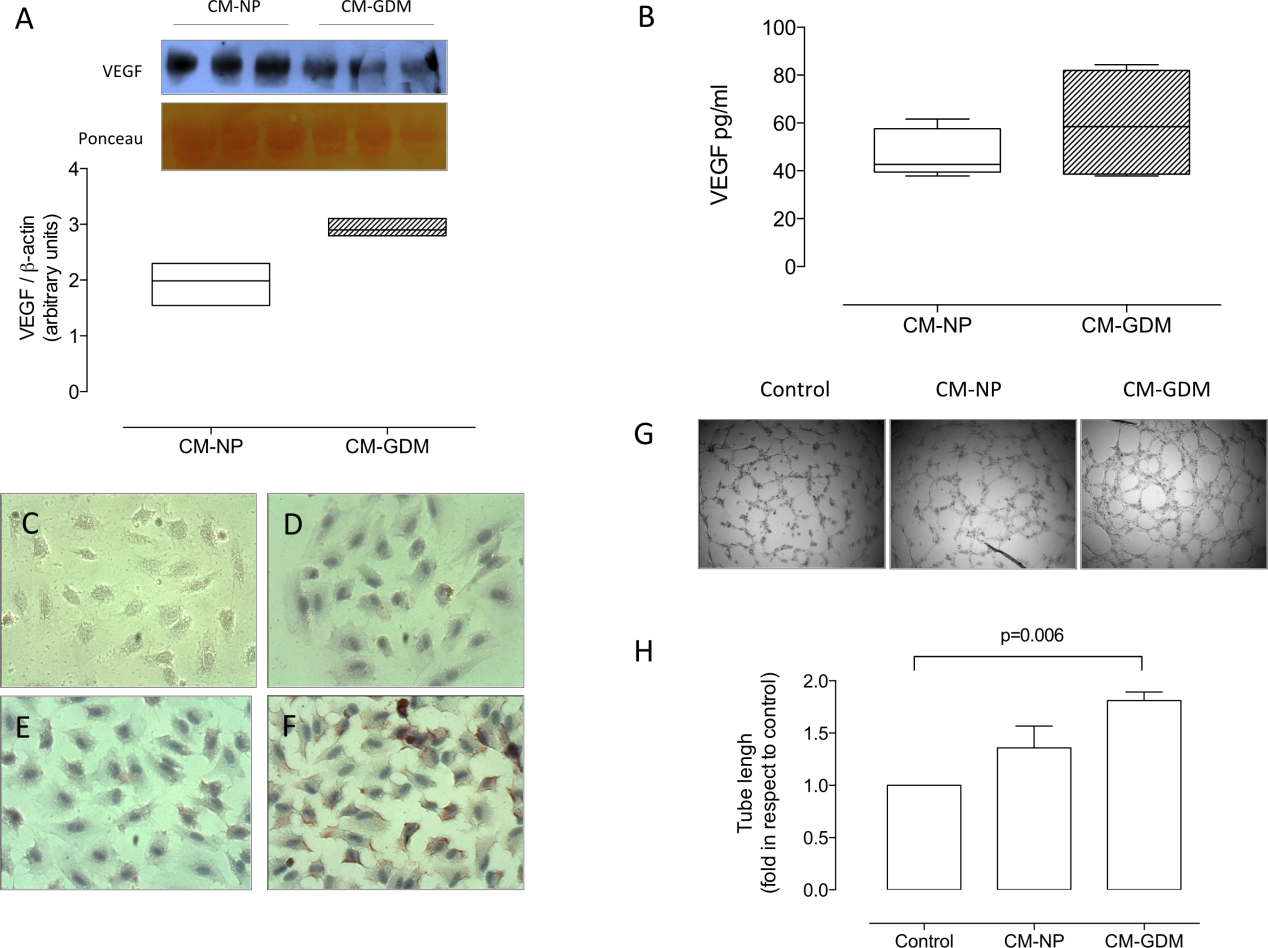
Characterization of conditioned medium from HUVEC isolated from GDM. **A)** Estimation of VEGF concentration by western blot in conditioned medium harvested from HUVEC derived from normal (CM-NP, white bar) or GDM (CM-GDM, hatched bar). **B)** Quantification of VEGF in conditioned medium using ELISA as in A. **C)** Representative images of negative control (without primary antibody) in the immunocytochemistry for KDR identification in HUVEC isolated from normal pregnancies and exposed (8h) to either **D)** M199 (control medium), **E)** CM-NP or **F)** CM-GDM. **G)** Tube formation assay in HUVEC in presence (8h) of control medium (control), CM-NP or CM-GDM. **H).** Tube length quantification in the tube formation assay. Magnification 40X. All Figs represent n = 3 in each group. Significant differences are indicated with respective P value.

Conditioned medium (CM) harvested from HUVEC from normal (CM-NP) or GDM (CM-GDM) pregnancy exhibited similar concentration of VEGF (Fig 6A and 6B), although a tendency (P = 0.07) to higher concentrations was observed in CM-GDM than CM-NP in western blot assays (Fig 6A).

In parallel experiments a significant increase (2.2- and 1.8-fold, respectively) in the relative amount of KDR protein (immunocytochemistry; Fig 6C–6F) and tube length (Matrigel assay; Fig 6G and 6F) was found in HUVECs isolated from normal pregnancies exposed (8h) to CM-GDM in comparison to untreated cells.

### CM-GDM induced proliferation is associated to Y951-phosphorylation of KDR

**Fig 7:**
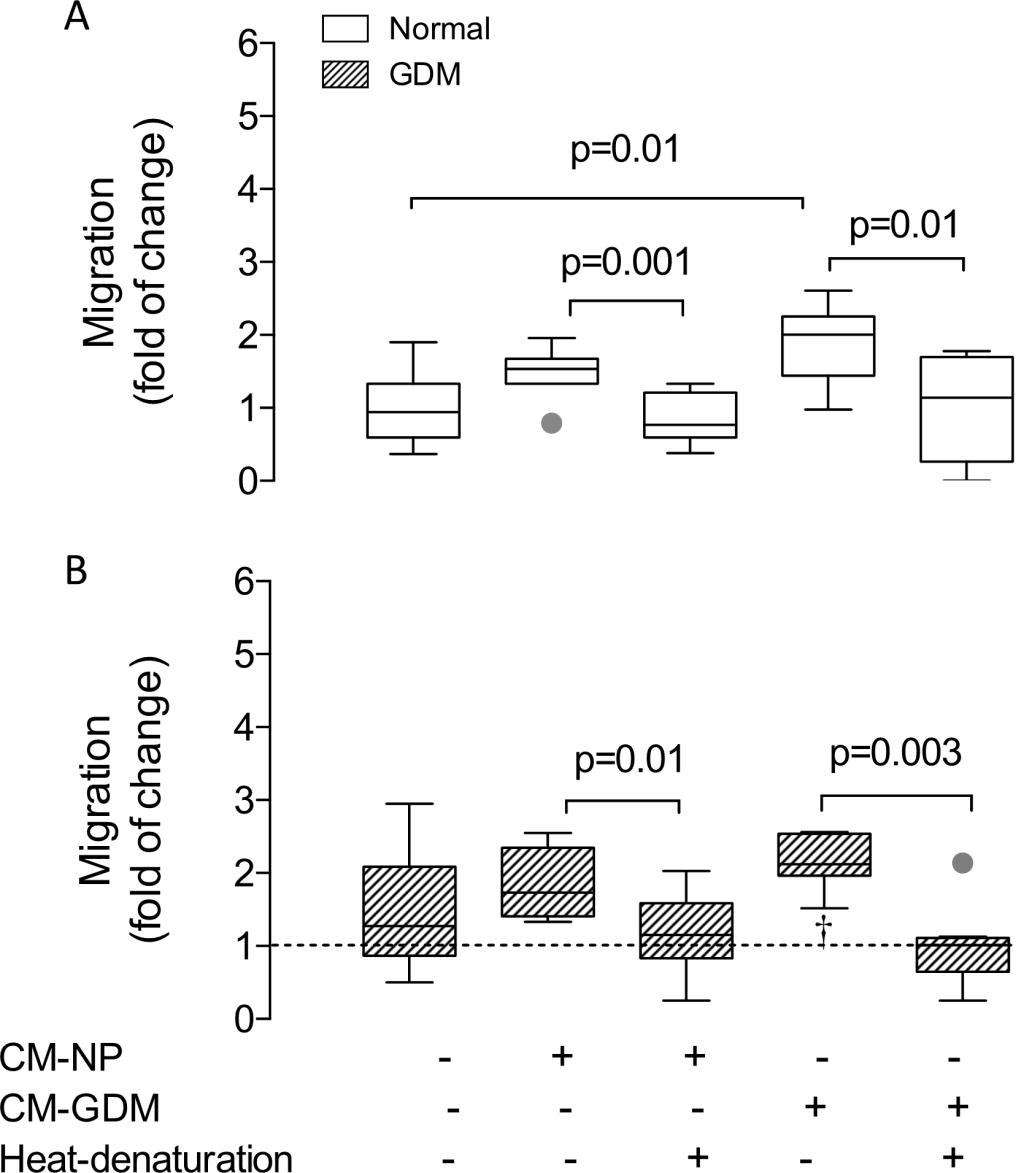
Cell migration in fetal endothelium from gestational diabetes mellitus. **A)** Cell migration (8 h) of HUVEC isolated from normal (Normal, white bar) or **B)** gestational diabetes mellitus (GDM, hatched bar) pregnancies exposed to conditioned medium harvested from cell cultures of either normal (CM-NP, +) or gestational diabetes mellitus (CM-GDM, +) pregnancies, or respective heat denaturized conditioned medium (heat denaturation). Representative images are available upon request. n = 4 in each group. Significant differences are indicated with respective P value. In B, line represents basal value in normal pregnancy. †P<0.05 vs HUVEC from normal pregnancy cultured in M199.

In parallel experiments cell migration was analyzed in the presence (8 h) of CM-GDM or CM-NP. HUVEC from normal pregnancy exposed to CM-GDM exhibited elevated migration than those cultured in M199 without serum (basal) or CM-NP (1.9 and 1.3 fold, respectively) (Fig 7A and 7B). This effect was abolished when CM-GDM was heat-denatured prior to use. Interestingly, HUVEC isolated from GDM did not exhibit any significant change when they were exposed to either CM-NP or CM-GDM. However, GDM cells exposed to heat-denatured CM-GDM showed reduced cell migration compared to basal condition or cell exposed to CM-GDM (-41% and -60%, respectively).

In addition, CM-NP and CM-GDM induced Y951-phosphorylation of KDR after 10 minutes incubation in HUVEC from normal pregnancy by 250% and 180%, respectively (S2B and S2C Fig), without significant changes in total KDR (S2A Fig). Y951-phosphorylation was abolished using heat-denatured CM (S2C and S2D Fig). No significant changes were observed in Y951-phosphorylation between CM-NP and CM-GDM, although a trend to higher phosphorylation was observed in CM-GDM (P = 0.4). HUVEC from GDM exposed to either CM-NP or CM-GDM exhibited no significant changes on Y951-phosphorylation. Raw data presented in this manuscript is included in S1 File.

## Discussion

**Fig 8:**
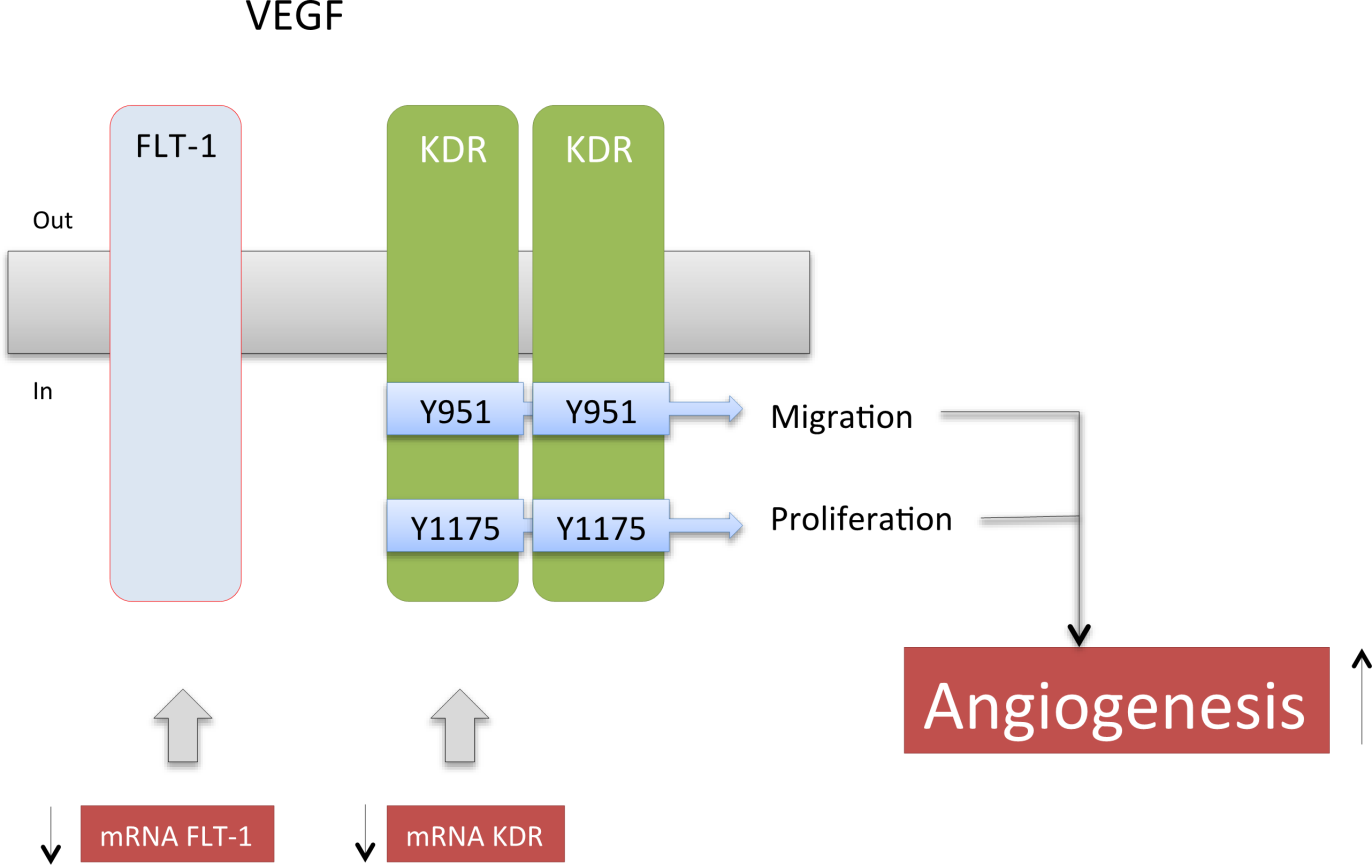
Working model. Results of this study suggest that placentas from gestational diabetes mellitus are in a proangiogenic state characterized by elevated endothelial cell markers, ie. more endothelial cells, and higher KDR, resulting in enhanced migration in GDM cells than cells from normal control pregnancies. Additional alteration/compensatory mechanisms include reduction in the mRNA and protein levels of Flt-1, reduced mRNA levels of KDR, and no changes in VEGF, or cell proliferation. Changes observed in GDM are shown in red and black arrows.

Collectively, the results of this study suggest that placentas from GDM pregnancies are in a proangiogenic state, since they have more endothelial cells i.e., elevated endothelial cell markers and placental blood vessels per chorionic villi, as well as higher level of total KDR than placentas from normal controls. However, we have to acknowledge that CD31 and CD34 are also present on hematopoietic progenitor cells, which may or may not contribute to hypervascularization in GDM [26]. Additional alterations associated with GDM include reduced Flt-1 expression (mRNA and protein), but no change of VEGF or KDR phosphorylation. In HUVEC no changes in proliferation markers were observed, but, interestingly, migration was higher in GDM cells than cells from normal control pregnancies. In line with this the conditioned medium harvested from GDM cell cultures enhanced the KDR amount and promoted cell migration and tube formation in HUVEC isolated from normoglycemic pregnancies after 8 hours of incubation. These results suggest that GDM is associated with high activation of KDR with ensuing overstimulation of endothelial cell migration. We speculate that this contributes to placental hypervascularization frequently observed in GDM pregnancies (see Fig 8).

In addition to placental angiogenesis, also the mean number of redundant capillary connections per terminal villus and the incidence of vascular hyperplasia (chorioangiosis) [16] is higher in GDM compared to controls suggesting a more pronounced capillary branching [27]. A hallmark of GDM is increased villous immaturity and increased measures of angiogenesis as recently reviewed by Huynh et al [9]. Our results also confirm increased placental angiogenesis in GDM.

The fetus vitally depends on adequate provision of oxygen. Fetal hyperinsulinemia in GDM stimulates fetal aerobic metabolism in GDM [28] and thus increases fetal oxygen demand. This occurs vis-a-vis elevated maternal levels of HbA1c, which has a higher affinity for oxygen than non-glycosylated HbA1. The resulting imbalance in oxygen supply and demand can lead to transient reduction of fetal oxygen levels (i.e., hypoxia), which together with elevated insulin are drivers for placental angiogenesis [29]. Thus, enhanced vascularization in GDM may represent a compensatory mechanism of the fetus to counteract permanent fetal hypoxia [30]. However, although the oxygen diffusive conductance is enhanced in GDM placentas [31], it is unclear whether placental hypervascularization in the GDM placenta is related to more oxygen delivery to the fetus. 3D-reconstruction of placental vasculature reveals that reduced vascular resistance due to high capillary sinuosity associated with capillary dilatation leads to effective oxygen uptake [32]. A different interpretation posits that a greater number of vessels in the villi can instead reduce oxygen diffusion to the fetus [21, 33].

Transient fetal hypoxia may trigger enhanced placental synthesis and release of VEGF, as well as the expression of VEGF receptors (i.e., Flt-1 and KDR) as reviewed previously by our group [6]. We have not measured cord blood VEGF concentrations in our collective, but other studies found reduced VEGF concentrations in umbilical cord blood from term fetuses born to mothers with GDM compared to healthy term fetuses [16, 18], while in placental tissue VEGF mRNA levels were not altered by GDM [17]. Our results show similar extracellular VEGF levels in CM-GDM and CM-NP.

While elevated VEGF levels do not seem to contribute to the proangiogenic state in GDM, changes at the receptor level either in the amount or activation or both may enhance VEGF effects. However, reports on VEGF receptor (i.e., Flt-1 or KDR) levels in human placentas in GDM are scant and vary between studies [17, 19, 34, 35]. Placentas from women with hyperglycemia contain high levels of VEGF and VEGF receptor 2 (KDR), but reduced expression of VEGF receptor 1 (Flt-1) compared to normoglycemic women [36]. Studies in pre-pregnancy diabetes also produced contradictory results [37–40]. This suggests that a range of unknown factors, including tissue sampling and clinical characteristics of the women, may have an effect on placental levels of VEGF and its receptors in GDM.

In the present study we have carefully considered mode of delivery and maternal pre-pregnancy BMI approximated by first trimester BMI. The results suggest that VEGF protein levels may not be significantly affected by BMI or delivery mode, whereas the GDM effect on Flt-1 protein levels depends on mode of delivery. It was significantly reduced only in women, who delivered vaginally, but not in the groups with cesarean section. Since Flt-1 has been considered as a decoy receptor of VEGF, we could speculate that reduced placental Flt-1 levels might constitute a compensatory mechanism in a proangiogenic diabetic scenario. Placental mRNA levels of Flt-1 are unchanged in GDM [17], but its soluble isoform sFlt-1 is elevated in placentas from GDM [41]. Despite these discrepancies it has been suggested that increased secretion and activity of VEGF may explain hypervascularization observed in placentas from GDM [6, 10, 16, 42]. However, the present study locates the relevant changes to the receptor level with a reduction in the expression of Flt-1, but increased KDR.

Activation of the KDR-mediated proangiogenic responses associated with phosphorylation of at least five tyrosine residues—951, 1054, 1059, 1175, and 1214 [12, 43]. Differential activation of KDR by VEGF has been previously described in endothelial cells [44]. For instance, Y951 appeared to be preferentially phosphorylated by matrix-bound VEGF, whereas Y1175 is preferentially phosphorylated by soluble VEGF [44]. In addition, the subsequent cell response depends on which site is phosphorylated. Thus, phosphorylation of Y951 of KDR has been associated with actin reorganization and cell migration [45], while phosphorylation of Y1175 leads to cell proliferation via the Src and ERK pathways [46]. This suggests that subtle differences in KDR phosphorylation at these sites might result in regulation of distinct, but different, cellular processes related to angiogenesis in GDM, similar to pre-eclamptic pregnancies [21].

Y1175-Phosphorylation of KDR was similar in placental homogenates from GDM and normal pregnancy. Importantly, HUVEC proliferation was also similar between these groups. In contrast, cell migration was higher in HUVEC from GDM than in controls. Also, HUVEC from normal pregnancies exposed to CM-GDM exhibited higher migration than cells exposed to CM-NP. The proangiogenic effect of CM-GDM was further supported by inducing (after 8 h) high KDR protein amount and tube formation in HUVEC from normal pregnancies. These results were associated with Y951-phosphorylation of KDR observed in cells exposed to CM-GDM or CM-NP, the former being slightly higher than the latter.

The angiogenic response to VEGF not only depends on its total concentration, but also on the spatial distribution and extracellular VEGF gradient [44, 47], as well as on the VEGF receptor location on the cell surface [12]. These factors lead to endothelial cell organization within vessels as tip and stalk cell phenotypes ultimately resulting in dynamic sprouting and branching angiogenesis [48]. Results showed more KDR in HUVECs from normal pregnancy exposed (8 h) to CM-GDM, associated with slight increase in Y951-phosphorylation of KDR in response to CM-GDM (10 min). Therefore, we suggest that CM-GDM induces KDR expression and/or activation leading to a higher migratory response.

We acknowledge few limitations of our study. We have tried to overcome the small cohort size by complementing *ex vivo* with *in vitro* studies. Analysis of a potential influence of newborn sex was precluded by the cohort size. Western blot analysis for Y951 residue of KDR was difficult to perform because of varying molecular size in the blots and a double band with estimated sizes of 70 kDa was clearly found. This bands at 70 kDa may represent immature forms of KDR a suggested by Takahashi and Shibuya [49]. We also did not further study KDR expression and focused on protein and functional analyses. This decision was also based on the discrepancy of KDR mRNA and protein levels i.e. lower mRNA; higher protein in GDM. Identifying the reason for these divergent KDR changes in GDM was outside the scope of our study, but likely involves microRNAs such as miR-199a-3p [50], miR-410-3p, miR-497-5p, and miR-2355-5p [51] or miR-16 [52] This warrants future studies. A regulation via microRNA would not be unexpected, since angiogenesis in general, and VEGFR expression in particular, is a dynamic process [13, 53]. Despite these limitations, this report suggests for the first time that differential activation of KDR may contribute to the proangiogenic state observed in placentas from GDM.

In conclusion, GDM features as a pro-angiogenic placental state. These results appear related to the reduced expression of Flt-1, but increased activity of KDR. Interestingly, KDR phosphorylation at Y951, but not at Y1175, was higher in placentas from GDM than normal pregnancy, suggesting a differential role for these phosphorylation sites in the underlying cellular mechanism accounting for the proangiogenic state observed in GDM. This results in stimulated cell migration rather cell proliferation. The implications of these findings to fetal well-being remain to be elucidated. Our knowledge regarding the roles of the VEGFRs is scant and warrants further studies.

## Supporting information

S1 Fig Post-hoc analysis of Flt-1 and KDR. Protein levels of Flt-1 (A) or KDR (B) as shown in Figs 3 and 4, respectively, were analyzed considering delivery mode in normal pregnancy (Normal, white bar) and gestational diabetes mellitus (GDM, hatched bar). Data is presented as densitometry of Flt-1 or KDR/β-actin ratio. In A, P value is presented in each analysis.
(TIFF)

S2 Fig Phosphorylation of Y951 of KDR in presence of CM. **A)** Representative images of total KDR (170kDa) and β-actin (43 kDa) in HUVEC isolated from normal (Normal, white bars) or gestational diabetes mellitus (GDM, hatched bars). Chart represents densitometry of KDR/β-actin ratio. **B)** Representative western blot of pY951 of KDR (70 kDa) in presence (5–15 min) of conditioned medium from normal (CM-NP) or GDM (CM-GDM). **C)** Representative images of phosphorylated Y951 of KDR (70-85kDa, pY951) and β-actin (43 kDa) in HUVEC isolated from normal or GDM. Also heat-denatured CM-NP or CM-GDM was used in parallel experiments. **D)** Densitometry of pY951KDR/β-actin ratio and in C. In A, C and D, n = 6 per group. In B, n = 2 per group. In D, *P<0.05 vs Basal in Normal. †P<0.05 vs HUVEC from normal pregnancy cultured in CM-NP.
(TIFF)

S1 File Excel file with the raw data. (XLSX)

## Abbreviations

GDM: Gestational diabetes mellitus
VEGF: Vascular endothelial growth factor
Flt-1: vascular endothelial growth factor receptor type 1
KDR: vascular endothelial growth factor receptor 2
Y951: Tyrosine 951
Y1175: Tyrosine 1175
p: phosphorylation
CM: conditioned medium
