# Supplementary figures and images for: Gestational diabetes mellitus is associated with increased pro-migratory activation of vascular endothelial growth factor receptor 2 and reduced expression of vascular endothelial growth factor receptor 1
Source: PLoS One. 2017 Aug 17;12(8):e0182509. doi: 10.1371/journal.pone.0182509 (PMC5560693; doi:10.1371/journal.pone.0182509)

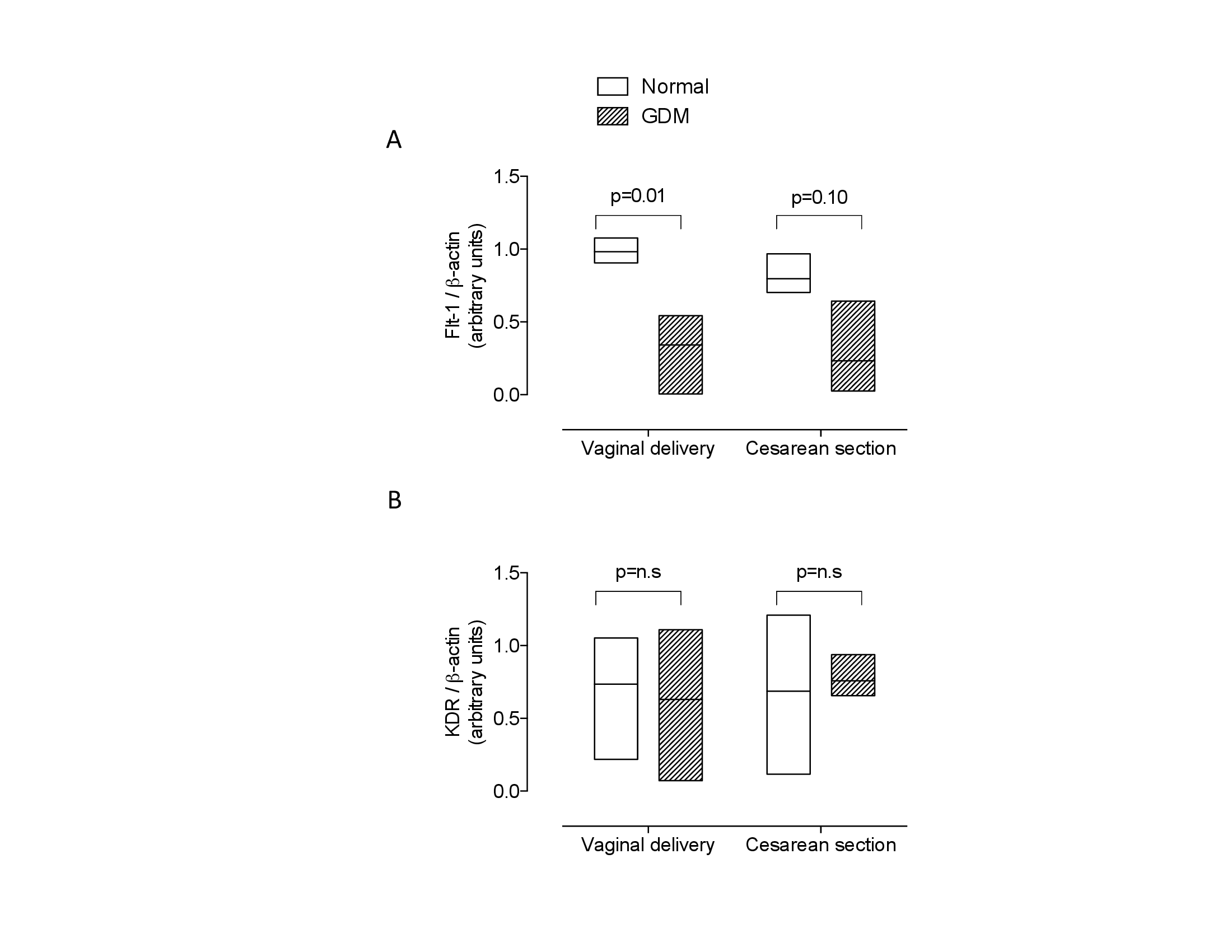

Supplement: S1 Fig — Protein levels of Flt-1 (A) or KDR (B) as shown in Figs 3 and 4, respectively, were analyzed considering delivery mode in normal pregnancy (Normal, white bar) and gestational diabetes mellitus (GDM, hatched bar). Data is presented as densitometry of Flt-1 or KDR/β-actin ratio. In A, P value is presented in each analysis. (TIFF) [file pone.0182509.s001.tiff]

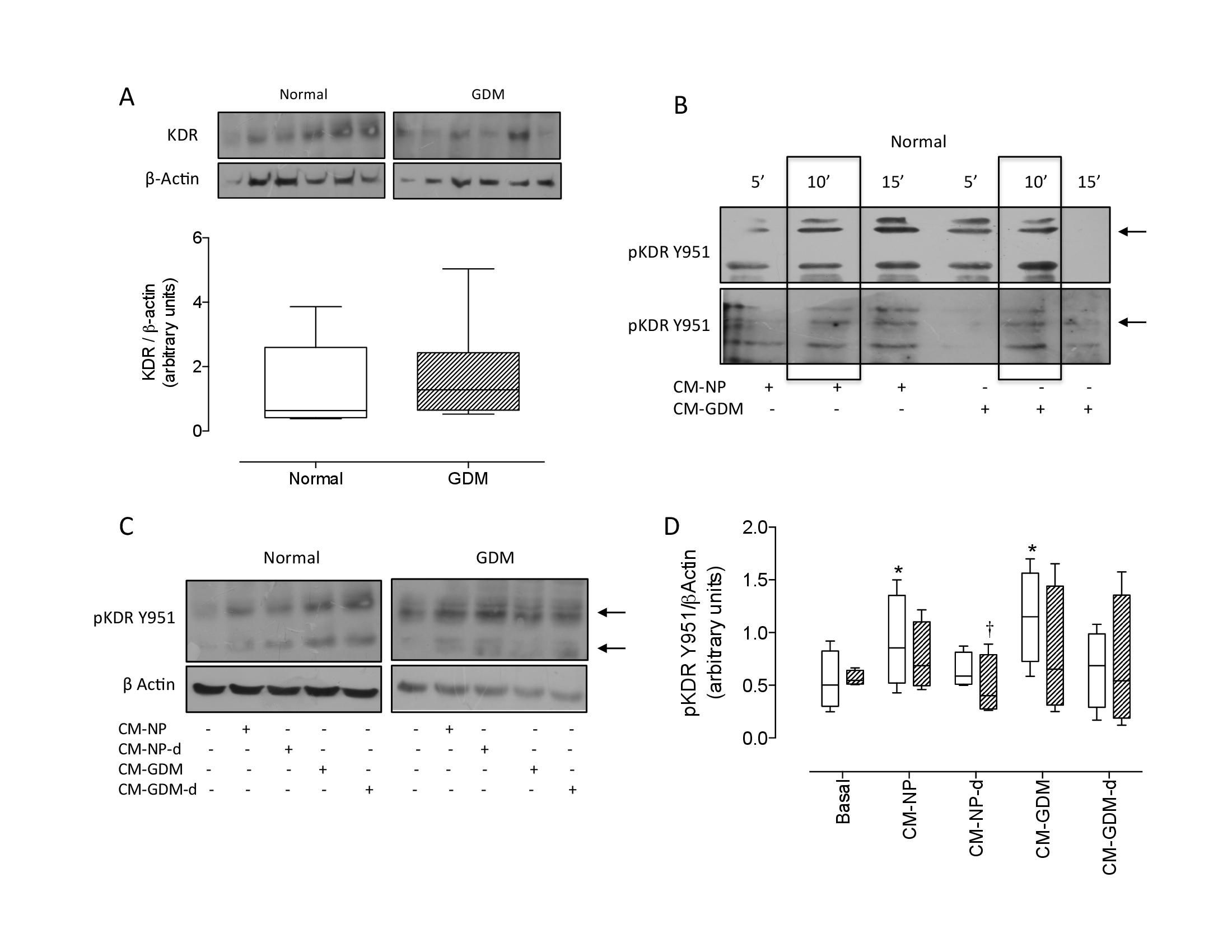

Supplement: S2 Fig — A) Representative images of total KDR (170kDa) and β-actin (43 kDa) in HUVEC isolated from normal (Normal, white bars) or gestational diabetes mellitus (GDM, hatched bars). Chart represents densitometry of KDR/β-actin ratio. B) Representative western blot of pY951 of KDR (70 kDa) in presence (5–15 min) of conditioned medium from normal (CM-NP) or GDM (CM-GDM). C) Representative images of phosphorylated Y951 of KDR (70-85kDa, pY951) and β-actin (43 kDa) in HUVEC isolated from normal or GDM. Also heat-denatured CM-NP or CM-GDM was used in parallel experiments. D) Densitometry of pY951KDR/β-actin ratio and in C. In A, C and D, n = 6 per group. In B, n = 2 per group. In D, *P<0.05 vs Basal in Normal. †P<0.05 vs HUVEC from normal pregnancy cultured in CM-NP. (TIFF) [file pone.0182509.s002.tiff]
